# Pennsylvania Hospital

**Published:** 1838-02-28

**Authors:** 


### CLINICAL LECTURES.

### PENNSYLVANIA HOSPITAL.

#### STRICTURE OF THE RECTUM.

*Wednesday, February 21st.*—DR. HARRIS commenced as follows: I propose, gentlemen, to offer some remarks this morning on strictures of the rectum, and will afterwards introduce a patient affected with this distressing disease.

Properly to understand the nature and treatment of this complaint, it is important that the surgeon should possess an accurate knowledge of the anatomy of the part involved.

The rectum commences at the sigmoid flexure of the colon, on the left side of the sacro-vertebral articulation, and descends obliquely to the right, in front of the sacrum, and accommodates itself to the curvature of this bone. It proceeds downwards under the prostate gland in the male, and the vagina in the female, where it is surrounded by cellular tissue, and terminates in the anus. Its various curvatures and folds should be held in recollection by the operator.

Stricture of the rectum, as in all other hollow tubes, is the result of irritation of this bowel. This may be from active purgation, stimulating injections, mechanical injuries inflicted on the intestine in difficult labours, ending in inflammation and thickening of the mucous and cellular coats. These strictures not unfrequently follow dysentery: I have seen three cases of stricture follow this disease.

This altered condition and thickening of the canal may occur at any point in the rectum, from the sigmoid flexure of the colon to the anus. In a majority of instances, however, it is found within three or four inches of the inferior part, but, in a case which I had under my care last summer, the morbid induration occupied three inches of the upper portion of the bowel. The disease had existed for seventeen years, and the caliber of the intestine was so contracted, as barely to admit a common sized urethra bougie. The induration in this case was so firm and unyielding, as to require three months completely to remove it. It was connected also with a stricture of the membranous part of the urethra. Bell, Copeland, White, Salmon, and others have recorded similar cases.

It is sometimes complicated with fistula in ano. In such cases, the internal aperture is immediately above the stricture. This is caused by the irritating substances, which lodge above the narrowed canal, causing inflammation and ulceration, which destroy the coats of the bowel at that point. The contents of the intestines may escape through the perforation and become extravasated into the cellular texture, which causes active inflammation, terminating in abscess. I was consulted about two years ago by Mr. W. of Union street, whose rectum was in this condition. The fistulous opening into the rectum was nearly an inch in diameter, so that when he had a fluid discharge from the bowels, (for he could have nothing else,) one half of it passed through the fistula, and the remainder through the natural channel. In females, ulceration from the same cause, may produce a perforation into the vagina.

The symptoms of stricture of the rectum, consist first of irregularity of the bowels, evacuations deficient in quantity, sometimes discharged in pellets, or flattened like tape; the bowels are costive; pain is felt in the diseased part, or about the anus, and occasional pains are felt in the loins, extending to the hips and thighs. As the disease becomes more aggravated, costiveness increases, to remedy which, under a supposition that his bowels are lethargic, the patient resorts constantly to purgatives. Those aperients which most successfully liquify the contents of the bowels, afford the most prompt relief. This condition of the rectum will sometimes continue for months and years. At last, this temporary treatment ceases to afford any permanent benefit.

After going to stool, the sensation is, as if the lower bowels were still distended, the disposition to go to stool continuing; the patient endeavours to force out the accumulated matter, but so ineffectually, that little except blood is discharged. When the stricture is near the anus, the efforts will sometimes produce a prolapsed state of the bowel. Days will frequently pass withont any evacuation, though the patient is continually annoyed by the most painful and ineffectual efforts. This confined state of the bowels is sometimes alternated with painful colicky diarrhœa.

Large accumulations of gas and fœcal matter distend the colon enormously, which is often followed by inflammation, and not unfrequently death. In less violent cases, the functions of the stomach become impaired; the bladder is affected with either retention or incontinence of urine, the kidneys secrete a fluid of a high colour, and of an offensive odour.

In the advanced stage of this disease the feet and legs are cold and cramped, accompanied by heat, fulness, and pain in the head; particucularly in the occipital portion of it. These symptoms are supposed to arise from the distended bowels pressing on the aorta, thus partially obstructing the flow of the blood to the lower extremities, and necessarily throwing an increased quantity to the head.

The nature of this disease being often misunderstood, it is neglected until the aggravated symptoms supervene. The patient is treated with aperients, until repeated alarming attacks convince him of his error.

The treatment of stricture of the rectum consists first, in allaying the inflammation of the part affected, by means of leeches to the anus, and cups to the perineum and loins, by a regulated diet, and by frequent injections of tepid water. In addition to the injections, mild aperient medicines should be daily exhibited. The following compound, taken in the evening, and followed by a warm water injection in the morning, will commonly produce an evacuation without irritation. The water should be retained in the bowels for the space of twenty minutes or more, in order that it may dissolve the hardened fecal matter. The following is the electuary to which I have alluded:

℞ Confectionis Sennæ, ℥i

Sulphuris Præcipitati, ʒiii

Olei Juniperi, gtt. vi m.

Of this take a tea spoonful every night.

While these general remedies palliate the symptoms, still we are to rely on the skilful use of bougies to accomplish a cure. It is highly important to have these instruments properly constructed. They should be made of such materials, that they may be readily adapted to the form of the rectum, otherwise serious injury may be inflicted on the diseased organ. The instrument which I have commonly used is made of waxed linen, and in the same way that the urethra bougie is formed. Salmon recommends that fine linen should be thickly coated with bees-wax, melted down with a small quantity of diachylon, and lamp-black. A piece of linen tape should be wrapped up in the cloth, extending more than half the distance of the instrument, and so arranged as to form a loop at its large end.

The largest sized bougie commonly used is about three inches in circumference, and the smallest about one inch. The two extremes should be divided into ten sizes, forming on the whole an increase from one to twelve. The length of the bougie must depend on the height of the patient. Where he is of the medium size, say five feet ten inches, the bougie should be about eleven inches. Mr. Evans, at the corner of Third and Spruce streets, makes them of the best form.

The flexible metallic and gum elastic bougies are by no means adapted to strictures, located at a distance from the anus. It is impossible properly to accommodate them to the varied curves of the rectum. In the hands of the experienced surgeon, they are inferior to wax bougies; in those of the inexperienced, they are highly dangerous.

An hour before you propose to introduce the bougie, an injection of a wine glass of flaxseed tea, with fifty or sixty drops of laudanum should be administered. The patient should be directed to pass off his urine, and then, if a male, to lean over a table, bend forward, separate the nates, and thus fairly expose the orifice of the bowel.

A full sized bougie, softened by immersion in warm water, well oiled, and shaped so as to conform to the curvatures of the intestine, is to be introduced, with the concavity of the instrument towards the sacrum. After it passes two and a half or three inches, the point of the instrument will bear on the hollow of the sacrum. In order to facilitate the passage of the instrument, we now change its position, by describing the segment of a circle by which the end of the instrument is brought forward, and, by the application of a gradual and moderate force, it is advanced to the upper part of the rectum. By depressing the but-end of the instrument, and propelling it onward, it enters the sigmoid flexure, and is fairly within the sphincter ani. While the instrument is passing, the patient frequently complains of cramps in the thighs, and pain in the region of the umbilicus. When the instrument meets an obstruction, pressure for a few minutes is to be maintained, and, if this pressure produces an aggravation of pain, without advancing, it is to be withdrawn, and a smaller size introduced, with the same precautions. If this should fail also, a smaller and smaller must be employed, until it enters, without much pain, the whole extent of gut into the sigmoid flexure. Unless the bougie has reached this point, it will recoil as soon as the pressure of the fingers is removed.

The instrument should remain within the stricture from ten minutes to half an hour, according to the irritability of the part. When the bougie is removed, it should harden into the form which it possessed when withdrawn. This will be of service in the subsequent introductions, as it will form a model for the shape of the other instruments, which may be used during the curative process. During the first introductions, a strong desire will be awakened to go to stool, which, however, is not felt in subsequent operations. Copeland, White, and others recommend the daily introduction of the bougie until a cure is accomplished. In a majority of cases, this frequency produces too much pain and irritation. My own experience has taught me, that from two to five days is as often as the patient can endure the bougie with advantage. The same sized instrument should be used two or three times, before we should resort to a larger one. It is difficult, it is true, to establish rules in such cases, as the surgeon must be governed by the extent and nature of the contraction, and the irritability of the diseased part.

The bougie acts on the principle of pressure, and thus causes an absorption of the altered and indurated structure. If the pressure can be sustained for several hours at a time, without giving rise to irritation, the sooner will the cure be affected. When, however, it does provoke inflammation, mischief, instead of benefit, results. The rule then should be, not to repeat the use of the bougie so long as the irritating effects of a previous operation, have not subsided.

After the introduction of the bougie, severe cramps of the lower extremities will sometimes occur, accompanied by rigors, and sickness of the stomach. When the instrument is forced into the sigmoid flexure of the colon, and while it remains there, pains are sometimes felt, resembling those which are experienced from medicine. After a few introductions of the bougie, these symptoms will cease to occur. When the instrument is withdrawn, the patient will sometimes discharge a large quantity of a jelly-like substance, possessing an odour extremely offensive.

It has been remarked, that, during the process of treatment, patients are very liable to be affected with catarrh. This circumstance has been particularly noticed in several excellent monographs on this disease.

In many instances, the bowels have their regular evacuations even before the stricture is entirely removed. In other instances, they remain a long time inactive, and require the frequent use of mild aperents or injections.

The use of the bougie should be continued until the stricture is enlarged to the natural size of the intestine, and it should be afterwards occasionally introduced, at distant intervals, for at least a year. Before the surgeon relinquishes his attentions to the case, he should teach the patient to use the bougie himself, and thus place any subsequent contraction under his own management.

The division of the stricture by cutting instruments, or the destruction of it by caustic, has been frequently recommended. As there is more or less danger incurred by these methods, they should be abandoned, and the milder and more effectual plan already indicated should be adopted. There is no case requiring the knife, except when stricture is complicated with fistula in ano. Under such circumstances, the intervening solid parts, including the stricture, must be divided.

[The patient was now placed in a proper position, and the second sized instrument was introduced, with some difficulty, into the sigmoid flexure. The stricture in this case was located about three inches above the anus. The patient complained of some sickness, with spasms about the umbilicus.

Dr. Harris introduced a second patient with incomplete fistula in ano, on whom he operated in the usual manner.]

